# Investigation of cyclic peptides as drug delivery systems for the delivery of the anti-tuberculosis drug pyrazinamide

**DOI:** 10.1039/d5na01168j

**Published:** 2026-05-23

**Authors:** Batoul Makiabadi, Fereshteh Naderi, Mohammad Zakarianezhad, Aria Raessi

**Affiliations:** a Department of Chemical Engineering, Sirjan University of Technology Sirjan Iran; b Department of Chemistry, ShQ.C., Islamic Azad University Shahr-e Qods Iran fnaderi@iau.ac.ir; c Department of Chemistry, Payame Noor University (pnu) P. O. Box 19395-4697 Tehran Iran; d Faculty of Medicine, University of Debrecen 4032 Debrecen Hungary

## Abstract

One of the important goals of drug delivery in the treatment of diseases is to effectively deliver drugs to deep and inaccessible areas of tissues. In recent years, cyclic peptides (CPs) have been used as drug delivery systems due to their high affinity for their targets, stability against degradation, and low toxicity. In this study, the interaction of the anti-tuberculosis drug pyrazinamide (PY) with cyclic decapeptides of glycine, alanine, and serine and their binary alternating sequences was investigated at the M06-2X/6-31G(d,p) level of theory in the gas phase. Interaction energies, structural parameters, topological properties, and RDG, ELF, and IGM analyses were used to assess the strength of interactions in the complexes. The electronic properties of cyclic peptides were investigated and compared before and after the complexation process. Based on the findings of this study, cyclic peptides based on binary alternating sequences have a higher tendency to interact with the pyrazinamide molecule. Therefore, the use of a combination of amino acids in cyclic peptides allowed for the rational design of a new material with more favorable properties. These findings provide insights into the development of more effective drugs using cyclic peptides.

## Abstract 2

The interaction of cyclic peptides with pyrazinamide holds promise for their use as stable and biocompatible carriers for controlled drug delivery in the treatment of tuberculosis.

## Graphical abstract

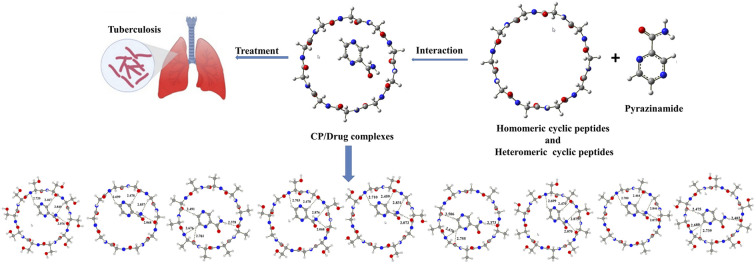

## Introduction

The delivery of therapeutic drugs to specific cells is a fundamental issue for the treatment of various human diseases, especially infectious, genetic, and cancer diseases. Developing drug delivery systems to penetrate and deliver drugs to target cells is a suitable solution for effective treatment of diseases.^1–7^ Drug delivery systems are materials that prevent drug degradation, increase its effectiveness, reduce its side effects, and control its release at the desired site.^8–11^ Recently, many studies have been conducted on the various properties of cyclic peptides and their role as drug carriers.^12,13^ Cyclic peptides are polypeptide chains formed by the connection of the amino terminus and carboxyl group of the chain, forming cyclic structures. Compared to linear peptides, cyclic peptides show greater potential for biological activities, due to a stable shape and state resulting from their cyclic structure.^14–17^ Cyclic peptides have a high affinity for binding to target tissue due to their large surface area. Due to their cyclic nature, these structures have less flexibility and more rigidity, and are therefore more stable.^18–21^ By changing the number and type of amino acids in cyclic peptides, the cyclic peptides can be modified as drug delivery systems.^22–24^ Wang and colleagues investigated the physicochemical properties of various types of cyclic peptides as drug delivery systems.^25^ Fakhari and co-workers reported that the cyclic peptides cyclo[(Ser–Ser)_4_], cyclo[(Gly–Gly)_4_], and cyclo[(Ala–Ala)_4_] could be effective carriers for the drug metformin.^26^ A theoretical study was conducted on the properties of cyclooctaglycine as a carrier for the anti-cancer drug penicillamine.^27^

Given the global threat of infectious diseases and the need for effective drug delivery, tuberculosis one of the deadliest infectious diseases worldwide.^28,29^ The most common types of drugs used to treat tuberculosis are rifampin, pyrazinamide, and streptomycin. Among the drugs used, pyrazinamide (PY) has shown good performance in treating patients with tuberculosis, by reducing side effects (fever, anorexia, liver enlargement, jaundice, and liver failure) and treatment duration.^30,31^ However, current treatment methods have limited effectiveness due to poor patient compliance with the drug regimen or due to the presence of drug-resistant tuberculosis. In recent years, studies have been conducted on the delivery of anti-tuberculosis drugs.^32–36^ Research shows that anti-tuberculosis drugs can be encapsulated in liposomes, microparticles, or nanoparticles for controlled entry and release into lung cells.^37–39^ The use of these therapeutic methods in infected animals shows a significant treatment improvement and reduction in complications and tissue damage.^40,41^ In addition to lipid-based and polymeric systems, various nanostructures such as carbon nanotubes (CNTs), fullerenes, boron–nitride nanotubes (BNNTs), and cyclic peptides have been widely investigated as carriers for pyrazinamide.^31,42–44^ Although CNTs and BNNTs have high mechanical strength and thermal stability, cyclic peptides have often attracted more attention for drug delivery applications due to their biodegradability, low toxicity, and ease of chemical modification through sequence engineering. Cyclic peptides have the ability to precisely tune the chemistry of the internal cavity by changing the amino acid sequence. This ability to tune the sequence is the main motivation for the present study.

Therefore, in this project, the interaction of the anti-tuberculosis drug PY with cyclic decapeptides of glycine, alanine, and serine and their binary alternating sequences was studied. Studying how drugs bind to cyclic peptides could lead to the design of molecules with higher affinity and fewer side effects. In this study, the complexes resulting from the interaction of PY with a variety of cyclic peptides were investigated. In this regard, structural parameters, interaction energies, atomic charge distribution, energy gap, electrostatic potential levels, charge transfer, and interactions strength were analyzed. This study attempts to answer the fundamental question, “What effect does changing the amino acid sequence in cyclic peptides have on the interaction with the drug pyrazinamide?” Therefore, in this work, we answer this question by systematically changing the CP sequence. By changing the amino acid sequence in cyclic peptides, an understanding of the structure–function relationship can be achieved, which allows for the rational design of drug carriers based on sequence chemistry. These insights are inaccessible when only a single sequence is studied. It is hoped that this study will provide a better understanding of how drugs bind and the role of the cyclic peptide structure, which could lead to the design of drugs with better absorption in the body.

## Computational methods

Density functional theory (DFT) was used to investigate the interaction of the drug PY with a number of cyclic decapeptides made of alanine, glycine, and serine amino acids. All structures were optimized at the M06-2X/6-31G(d,p) level of theory using the Gaussian 09 software package.^45^ The counterpoise procedure (CP)^46^ was used to correct for basis set superposition error (BSSE) in the calculation of different binding energies. The interaction energy (Δ*E*_ads_) is calculated as:

1 Δ*E*_int_ = *E*_(Comp)_ − (*E*_(PY)_ + *E*_(CP)_)

where *E*_(Comp)_ is the total energy of the complex of the drug interacting with the cyclic peptide, *E*_(PY)_ is the total energy of a drug molecule, and *E*_(CP)_ is the total energy of a cyclic peptide. For all complexes, the molecular descriptors such as the HOMO–LUMO energy gap (*E*_gap_), hardness (*η*), softness (*S*), electrophilicity index (*ω*) and the maximum amount of electronic charge (Δ*N*_max_) were calculated as:

2 *E*_gap_ = *E*_LUMO_ − *E*_HOMO_

3 *η*= ((*E*_g_)/2)

4 *S* = 1/(2*η*)

5 *ω*= (*µ*^2^/2*η*)

6 Δ*N*_max_ = −*µ*/*η*

When two systems A and B approach each other, the amount of charge transfer between them can be written in terms of electrophilicity. Electrophilicity-based charge transfer (ECT) is obtained by:

7 ECT= (Δ*N*_max_)_A_ − (Δ*N*_max_)_B_

where ECT > 0 then A is an electron acceptor, while if ECT < 0 it is an electron donor.^47^

The solvent effect was examined using the M06-2X/6-31G(d,p) level of theory by applying the polarizable continuum model (PCM).^48^ The Multiwfn program was used to plot the electron density of states (DOS), the electron localization function (ELF),^49^ and independent gradient model (IGM). The reduced density gradient (RDG) plots were rendered by the VMD program^50^ based on the outputs of Multiwfn. The NBO and AIM analysis was carried out at the M06-2X/6-31G(d,p) level of theory.^51,52^

## Results and discussion

### Energies and geometries

**Scheme 1:**
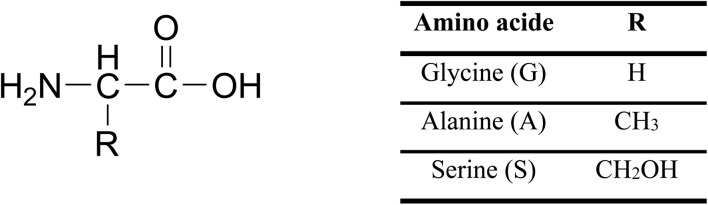
The linear structure of the amino acids glycine, alanine, and serine.

DFT calculations were performed to evaluate the interaction of the PY drug with a number of cyclic peptides. The difference between these cyclic peptides is in the type and alternating sequence of amino acids used in them. In this regard, three types of amino acids were selected, such as alanine, glycine, and serine molecules (Scheme 1).

**Fig. 1:**
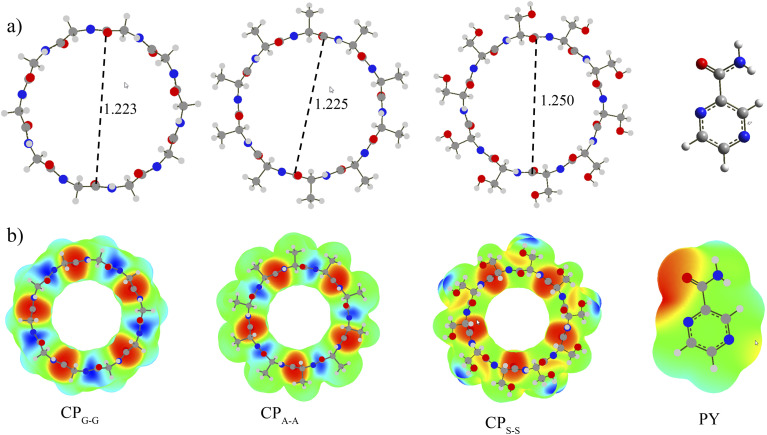
(a) The optimized structures; (b) the molecular electrostatic potential surface maps for CP_G–G_, CP_A–A_, CP_S–S_, and PY structures.

In the first stage, cyclic peptides composed of one type of amino acid were designed. Using 10 molecules of each, cyclic structures CP_X–X_ (X = S, G, and A) were constructed and named CP_G–G_, CP_A–A_, and CP_S–S_ (see Fig. 1).

As seen in the optimized structures, the oxygen atoms of the ketone groups are located alternately up and down of the cyclic peptides. It is observed that the length of C=O, C–C, and C–N bonds increases with the change in R group from hydrogen to methanol. Therefore, it is observed that the length of C–N bonds depends on the R group. The ring diameter from the carbon of the carbonyl group to the carbon of the opposite carbonyl group in CP_G–G_, CP_A–A_, and CP_S–S_ is 1.223, 1.225, and 1.250 Å, respectively. The MESP (molecular electrostatic potential surface) maps were drawn for the drug and different cyclic peptides (Fig. 1). These maps were used to identify suitable sites on the drug and cyclic peptide for interaction with each other. In these color maps, red, blue, and green colors correspond to areas with negative, positive, and zero electrostatic potential, respectively. As can be seen, the oxygen atoms of the drug and the cyclic peptides have negative ESP, while the hydrogen atoms connected to N atoms have positive MESP. Therefore, the negative charges were placed on O atoms, while the positive charges were placed on H atoms. In CP_S–S_, CP_G–G_, CP_A–A_, and PY structures, the average electrostatic potential (ESP) on the local surface of the O atom of the C=O group is observed to have values of −19.8, −22.5, −31.5, and −32.7 kcal mol^−1^, respectively. Therefore, these sites are susceptible to electrophilic attack. In contrast, the average ESP on the local surface of hydrogen atoms is positive, and thus nucleophilic reagents tend to be attracted to these sites.

**Fig. 2:**
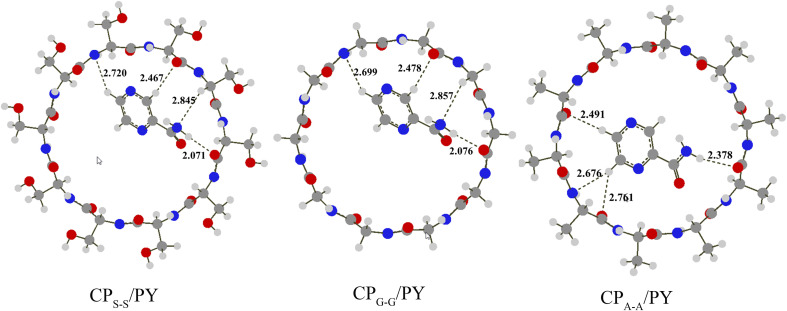
The optimized structures for CP_X–X_/PY complexes at the M06-2X/6-31G(d,p) level of theory.

In the first step, the DFT calculations were performed to evaluate the interaction of the PY drug with CP_S–S_, CP_G–G_, and CP_A–A_ cyclic peptides. The most stable structures were named CP_S–S_/PY, CP_G–G_/PY, and CP_A–A_/PY complexes (see Fig. 2). Two types of hydrogen bonds, O⋯H and N⋯H, are observed in these complexes.

Analysis of structural parameters in CP_S–S_/PY and CP_G–G_/PY complexes shows that in the interaction between the drug and the cyclic peptide, two O⋯H hydrogen bonds and two N⋯H hydrogen bonds are formed, while in the CP_A–A_/PY complex, three O⋯H hydrogen bonds and one H⋯N hydrogen bond are observed. Hydrogen bonds play important roles in biological systems.^53–55^ In the CP_X–X_/PY complexes, the lengths of the O⋯H hydrogen bonds are shorter than the N⋯H hydrogen bonds. These data suggest that O⋯H hydrogen bonding interactions are stronger than N⋯H interactions. On the other hand, the O⋯H hydrogen bond distances in the CP_S–S_/PY complex are shorter than those in the CP_G–G_/PY and CP_A–A_/PY complexes. It is predicted that the O⋯H hydrogen bonding interactions in the CP_S–S_/PY complex are stronger than those in the CP_G–G_/PY and CP_A–A_/PY complexes.

**Fig. 3:**
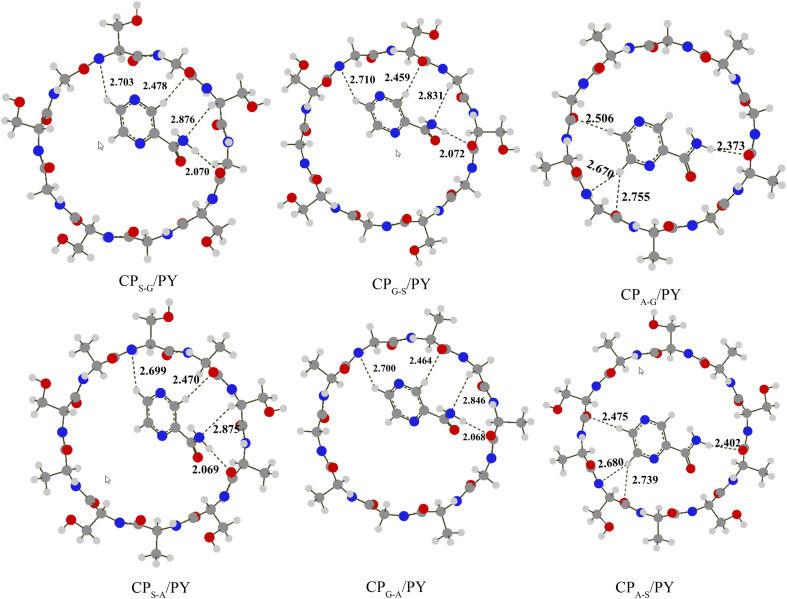
The optimized structures for CP_X–Y_/PY complexes at the M06-2X/6-31G(d,p) level of theory.

Studies show that the orientation of functional groups affects the spatial structure, stability, biological activity, and physicochemical properties of cyclic peptides. Therefore, the sequence of amino acids in the structure of cyclic peptides is of particular importance. To investigate this issue, the cyclic structures CP_X–Y_/PY(X = S, G, A and Y=S, G, A, X ≠ Y) were considered. In these structures, the amino acids serine, glycine, and alanine were alternately substituted in CP_X–X_/PY complexes. The resulting structures were named CP_S–A_/PY, CP_S–G_/PY, CP_G–S_/PY, CP_G–A_/PY, CP_A–G_/PY, CP_A–S_/PY, CP_S–S_/PY, CP_G–G_/PY, and CP_A–A_/PY complexes. The CP_X–Y_/PY optimized complexes at the M06-2X/6-31G(d,p) level of theory are shown in Fig. 3.

The number and type of hydrogen bonds observed in CP_X–Y_/PY complexes are similar to those in CP_X–X_/PY complexes. Also, in these complexes, the drug is closer to the cyclic peptide from the NH_2_CO side. The shortest hydrogen bond distance is 2.068 Å in CP_S–G_/PY, 2.070 Å in CP_S–A_/PY, 2.072 Å in CP_G–S_/PY, 2.075 Å in CP_G–A_/PY, 2.373 Å in CP_A–S_/PY and 2.402 Å in CP_A–G_/PY. This distance appears (O⋯H hydrogen bond) to be shorter and stronger in CP_X–Y_/PY complexes than in CP_X–X_/PY complexes.

The interaction energies (Δ*E*_int_) for CP_X–X_/PY and CP_X–Y_/PY complexes at two theoretical levels are summarized in Table 1. Based on Table 1, the relative stability of complexes decreases in the order CP_S–G_/PY > CP_S–A_/PY > CP_G–S_/PY > CP_G–A_/PY > CP_A–G_/PY > CP_A–S_/PY > CP_S–S_/PY > CP_G–G_/PY > CP_A–A_/PY. The results show that the Δ*E*_int_ values for CP_X–Y_/PY complexes are more than those for CP_X–X_/PY complexes. Therefore, structures with more negative interaction energies are more stable. Also, the type and sequence of amino acids in cyclic peptides affect their stability. According to the results obtained, it can be concluded that CP_X–Y_/PY structures are more stable than CP_X–X_/PY complexes. It can be concluded that CP_X–Y_/PY complexes have a higher affinity for drug interaction than CP_X–X_/PY complexes. The interaction energy results show that the stability order of the complexes remained unchanged on changing the basis set.

**Table 1:** The interaction energies (Δ*E*_int_/kJ mol^−1^), the recovery time (*τ*), charge transfer, and the hydrogen bond energy (*E*_HB_, kcal mol^−1^) for all complexes

Structure	Δ*E*_int_	Δ*E*^bsse^_int_	*τ* (s)	CT (e)	*E*_HB_
CP_S–S_/PY	−88.74^a^ (−78.50)^b^	−63.04	3.1 × 10^3^	−0.00004	0.185
CP_G–G_/PY	−43.64 (−20.56)	−28.22	4.1 × 10^−5^	−0.00180	0.180
CP_A–A_/PY	−18.52 (−16.03)	−5.53	1.7 × 10^−9^	−0.00840	0.104
CP_S–G_/PY	−100.80 (−95.58)	−69.88	3.8 × 10^5^	−0.00186	0.195
CP_S–A_/PY	−90.29 (−89.41)	−59.63	5.2 × 10^3^	−0.00008	0.189
CP_G–S_/PY	−88.84 (−86.32)	−57.96	3.5 × 10^3^	−0.00192	0.187
CP_G–A_/PY	−88.31 (−79.05)	−57.45	2.4 × 10^3^	−0.00188	0.184
CP_A–G_/PY	−68.65 (−62.10)	−42.59	0.1 × 10^1^	−0.0094	0.102
CP_A–S_/PY	−67.63 (−60.75)	−41.70	6.7 × 10^2^	−0.00879	0.072

a Interaction energies at the M06-2X/6-31G(d,p) level of theory.

b Interaction energies at the M06-2X/6-311++G(d,p) level of theory.

The Espinosa–Molins–Lecomte (EML) equation (*E*_HB_ = 0.5 × *V*(*r*)) is a formula used to estimate the energy of hydrogen bonds, where *V*(*r*) is the value of local potential energy at the bond critical point.^56^ The absolute value of *E*_HB_ (|*E*_HB_|) for the shortest hydrogen bond (O⋯H) is reported in Table 1. The *E*_HB_(|*E*_HB_|) value depends on the length of the hydrogen bond, so the shorter bonds have more (|*E*_HB_|) and *vice versa*. In CP_X–Y_/PY complexes, the absolute value of *E*_HB_(|*E*_HB_|) related to the O⋯H hydrogen bond is greater than that of CP_X–X_/PY complexes. This result is consistent with the shorter and stronger O⋯H interaction in CP_X–Y_/PY complexes than in CP_X–X_/PY complexes. The recovery time (*τ*) is a critical parameter for gas sensors and drug delivery systems.^57^ The term “recovery time” refers to the time required for a drug molecule to dissociate from the substrate surface. The recovery time can be calculated using conventional transition state theory (*τ* = *ν*_0_^−1^ × exp^−Δ*E*_int_/*kT*^),^58^ where Δ*E*_int_ is the interaction energy, *T* is the ambient temperature (298.15 K), *k* is the Boltzmann constant, and *ν*_0_ is assumed to be 10^12^ Hz. We used this formula as a measure to compare the strength of a drug binding to the cyclic peptides. The calculated time is used solely to compare the relative strength of interactions in different structures. High interaction energy indicates a strong interaction between the drug and the cyclic peptide and can lead to a longer recovery time. The results in Table 1 show that the recovery time for CP_X–Y_/PY complexes is longer than that of CP_X–X_/PY complexes, which is in agreement with the greater interaction energy of CP_X–Y_/PY complexes than CP_X–X_/PY ones. The CP_X–Y_/PY complexes are more stable than CP_X–X_/PY complexes and hold the drug for a longer period of time, whereas CP_X–X_/PY complexes release the drug rapidly, making them suitable for use in sensors. Therefore, the CP_X–Y_/PY complexes are better for drug delivery due to their longer *τ*.

### The electronic properties of structures

**Fig. 4:**
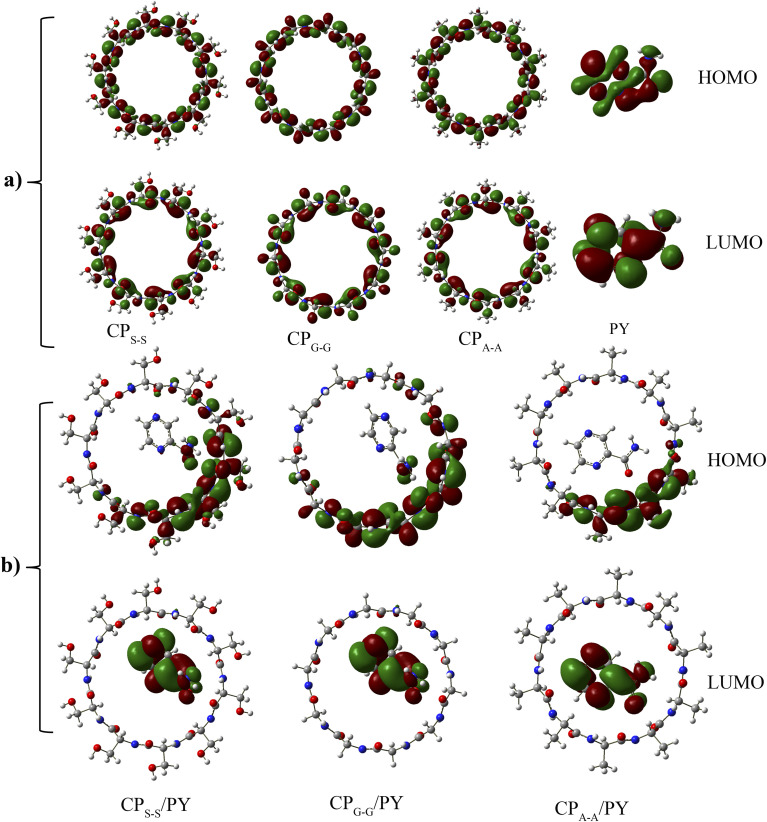
Charge distribution of the HOMO and LUMO for (a) monomers and (b) the CP_X–X_/PY complexes.

**Fig. 5:**
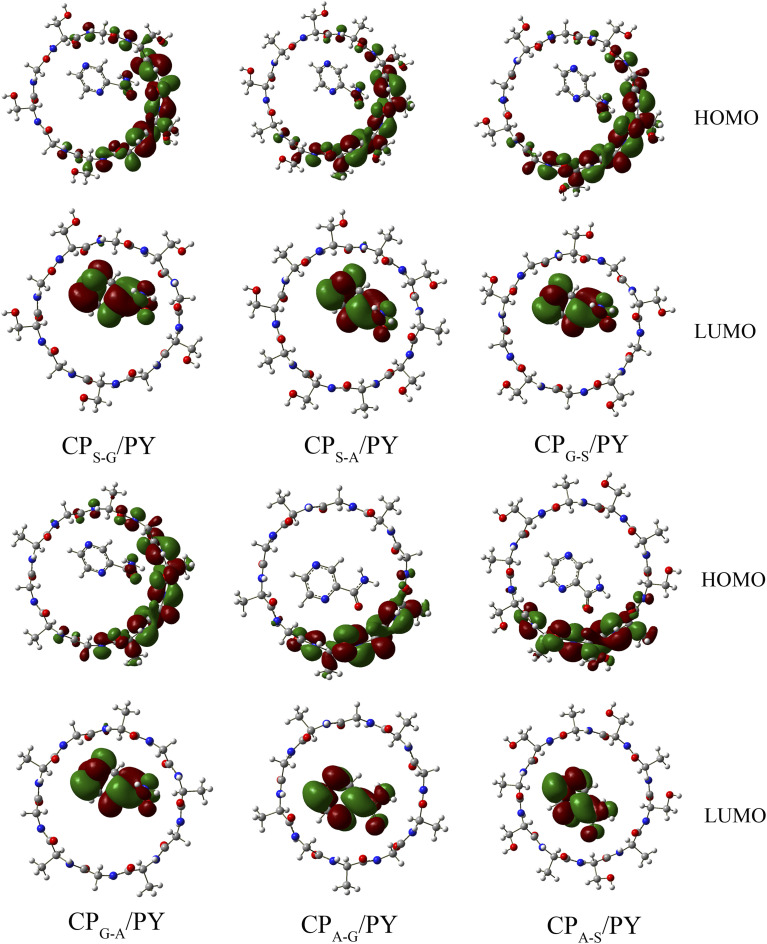
Charge distribution of the HOMO and LUMO for the CP_X–Y_/PY complexes.

The positions of the highest occupied molecular orbital (HOMO) and lowest unoccupied molecular orbital (LUMO) determine the electronic properties and reactivity of the structures. The HOMO and LUMO are referred to as electron donating and electron accepting orbitals, respectively. The charge distribution of the HOMO and LUMO for monomers and complexes is given in Fig. 4 and 5.

As can be seen, in all complexes, the electron density of the HOMO is distributed over the cyclic peptide and is mainly placed on the part involved in the interaction, while the LUMO is distributed on the drug. According to Fig. 4 and 5, the drug interacts with the HOMO of the cyclic peptide through its LUMO. It is predicted that cyclic peptides are more nucleophilic, whereas the drug molecule is electrophilic in nature. The stability of structures depends on the difference between the energy levels of the HOMO and LUMO. This difference is called the energy gap. Molecules with a higher energy gap are more stable than molecules with a lower energy gap. Some of the quantum molecular descriptors for monomers and complexes are given in Table 2.

**Table 2:** The molecular descriptors for monomers and complexes at the M06-2X/6-31G(d,p)level of theory in the gas phase

Structure	HOMO (eV)	LUMO (eV)	*E*_g_ (eV)	*η* (eV)	*S* (eV^−1^)	*µ* (eV)	*ω* (eV)	Δ*Ν*_max_ (au)	ECT (au)	*µ* (D)
PY	−8.557	−0.739	7.819	3.909	0.128	−4.648	2.763	0.707		3.41
CP_G–G_	−8.564	1.363	9.927	4.963	0.101	−3.601	1.306	0.263		0.00
CP_A–A_	−8.488	1.251	9.739	4.869	0.103	−3.618	1.344	0.276		0.00
CP_S–S_	−8.080	1.476	9.556	4.778	0.105	−3.302	1.141	0.239		0.00
CP_S–A_	−8.218	1.274	9.492	4.746	0.105	−3.472	1.270	0.268		1.80
CP_S–G_	−8.311	1.334	9.645	4.823	0.104	−3.489	1.262	0.262		0.54
CP_G–A_	−8.523	1.097	9.620	4.810	0.104	−3.713	1.433	0.298		0.42
CP_G–G_/PY	−8.446	−0.821	7.625	3.812	0.131	−4.634	2.816	0.739	0.476	2.73
CP_A–A_/PY	−7.880	−0.581	7.299	3.649	0.137	−4.230	2.452	0.672	0.404	3.57
CP_S–S_/PY	−7.958	−0.297	7.661	3.831	0.131	−4.128	2.224	0.581	0.342	2.45
CP_S–A_/PY	−8.131	−0.525	7.606	3.803	0.131	−4.328	2.463	0.648	0.380	2.77
CP_S–G_/PY	−8.174	−0.567	7.607	3.803	0.131	−4.371	2.511	0.660	0.399	2.88
CP_G–S_/PY	−8.186	−0.542	7.644	3.822	0.131	−4.364	2.492	0.652	0.390	2.92
CP_G–A_/PY	−8.379	−0.749	7.630	3.815	0.131	−4.564	2.730	0.716	0.418	2.78
CP_A–G_/PY	−8.132	−0.842	7.290	3.645	0.137	−4.487	2.762	0.758	0.460	3.37
CP_A–S_/PY	−8.071	−0.771	7.300	3.650	0.137	−4.421	2.677	0.734	0.457	3.37

**Fig. 6:**
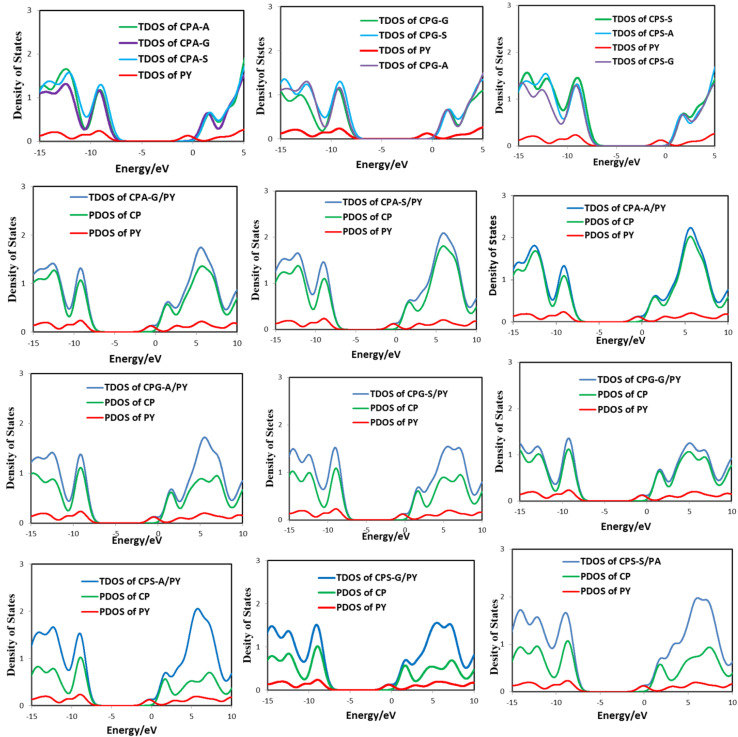
The total and projected density of states for CP_X–X_/PY and CP_X–Y_/PY complexes.

After the interaction of the PY with cyclic peptides, the HOMO and LUMO states move to lower and higher negative energies, respectively. Due to this change, the energy gap is reduced. This reduction in the energy gap can affect the fluorescence emission of the complexes, aiding in tracking the direction of the drug. The energy gap for CP_X–Y_ structures is lower than that of CP_X–X_ ones. It is predicted that CP_X–Y_/PY complexes are more reactive than CP_X–X_/PY complexes, with a greater tendency to interact with the drug. The results show that after complexation, the energy gap of CPs decreased, indicating that the interaction of the drug with CPs increases the reactivity of CPs. The energy gap values for complexes are decreased as follows: CP_X–X_/PY: CP_S–S_/PY > CP_G–G_/PY > CP_A–A_/PY; CP_X–Y_/PY: CP_G–S_/PY > CP_G–A_/PY > CP_S–G_/PY > CP_S–A_PY > CP_A–S_/PY > CP_A–G_/PY. The energy gap for CP_A–A_/PY and CP_S–S_/PY complexes is higher than that of their corresponding CP_X–Y_/PY complexes (CP_A–S_/PY, CP_A–G_/PY, CP_S–A_PY, and CP_S–G_/PY). Therefore, the reactivity of CP_A–A_/PY and CP_S–S_/PY complexes is less than that of their corresponding CP_X–Y_/PY complexes. This result is consistent with the lower interaction energy of CP_A–A_/PY and CP_S–S_/PY complexes compared to the corresponding P_X–Y_/PY complexes. The opposite of this result was observed in the CP_G–G_/PY complex. For further investigation, the total density of states (TDOS) and projected density of states (PDOS) diagrams for monomers and complexes are shown in Fig. 6. According to the plots, the LUMO and HOMO states shift towards more negative and lower values, respectively, which reduces the energy gap of CPs after interaction with the drug. In these diagrams, CPs and PY have the highest and lowest contributions, respectively.

The reactivity of chemical species could be associated with molecular descriptors such as electronic chemical potential (*µ*), hardness (*η*), softness (*S*), electrophilicity index (*ω*), dipole moment (*µ*), and the maximum charge transfer (Δ*N*_max_) (Table 2). As can be seen, these molecular descriptors changed upon complexation. In CP_X–X_/PY and CP_X–Y_/PY complexes, the value of *η* decreases, whereas the value of *S*, *ω*, *µ*, and Δ*N*_max_ increases upon complexation. Comparison of molecular descriptors in all complexes shows that the values of *E*_g_ and *η* for CP_A–A_/PY and CP_S–S_/PY complexes is more than that for their corresponding CP_X–Y_/PY complexes (CP_A–S_/PY, CP_A–G_/PY, CP_S–A_PY, and CP_S–G_/PY), in contrast, the values of *S*, *ω*, and Δ*N*_max_ for CP_A–A_/PY and CP_S–S_/PY complexes is less than that for their corresponding CP_X–Y_/PY complexes. It is predicted that the affinity of CP_A–A_/PY and CP_S–S_/PY complexes to the PY drug is less than that of their corresponding CP_X–Y_/PY complexes. The opposite of this result was observed in the CP_G–G_/PY complex. From Table 2, the dipole moment of CPs increases upon complexation. The dipole moment of the CP_X–Y_/PY complexes is more than that in CP_X–X_/PY complexes. Therefore, cyclic peptides with alternating sequences show a higher affinity for drug interaction in the gas phase. It is predicted that, in a polar solvent, the solubility of CP_X–Y_/PY complexes is higher than that of CP_X–X_/PY complexes.

### Solvent effects

Given the vital role of water in the human body, this substance was chosen as the solvent to study the behavior of CP/PY complexes under conditions similar to the biological environment. The interaction energies (Δ*E*_sln_), Gibbs free energies of solvation 
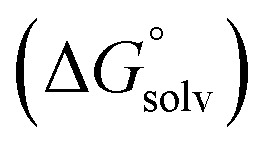
, the energy gap, electronic chemical potential (*µ*), hardness (*η*), softness (*S*), Δ*N*_max_ and dipole moment for the most stable complexes in water solvent are given in Table 3. All calculations were performed at the M06-2X/6-31G(d,p) level of theory. According to the results, the interaction energies (Δ*E*_sln_) of complexes have decreased in water solvent. The Δ*E*_sln_ value for the CP_S–S_/PY complex is lower than that of their corresponding CP_X–Y_/PY complexes, indicating that in the solution phase, the affinity of CP_S–A_/PY and CP_S–G_/PY complexes to the PY drug is higher than that of the CP_S–S_/PY complex. The stability order of the CP_X–Y_/PY complexes is almost similar to that of the gas phase. The negative values of 
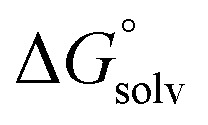
 show that the solvation process is spontaneous. Therefore, the solubility of the system is increased upon complexation. The 
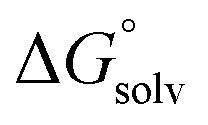
 can be separated into: 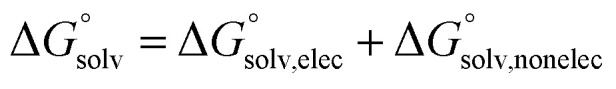. From Table 3, the contribution of the electrostatic component of 
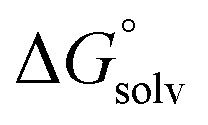
 is greater than that of the non-electrostatic component for complexes. Therefore, the electrostatic interactions between the solvent and the solute can lead to changes in the relative energies of the species in water.

The electrostatic 
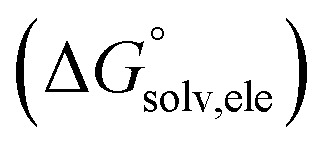
 and non-electrostatic 
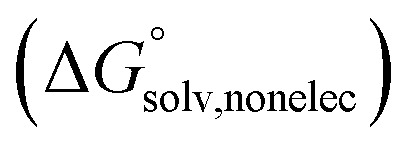
 contributions to the Gibbs free energy of solvation 
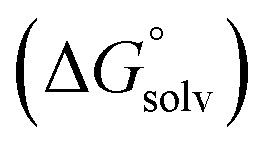
, the interaction energy in solution (Δ*E*_sln_), dipole moment (*D*), and the molecular descriptors for most stable complexes at the M06-2X/6-31G(d,p) level of theory in the solution phase

**Table:** 

Structure	Δ*E*_Sln_	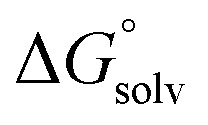	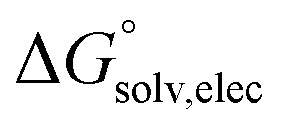	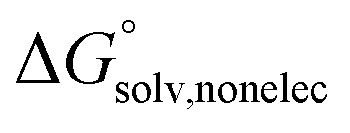	*µ* (D)	*E*_g_ (eV)
CP_S–S_/PY	−55.97	−62.48	−83.5	21.02	3.25	7.858
CP_S–G_/PY	−78.64	−80.73	−96.61	15.88	7.83	7.728
CP_S–A_/PY	−62.59	−77.71	−97.62	19.91	5.23	7.724

**Table:** 

Structure	*η* (eV)	*S* (eV^−1^)	*µ* (eV)	*ω* (eV)	Δ*Ν*_max_ (au)
CP_S–S_/PY	3.929	0.12726	−4.622	2.719	0.692
CP_S–G_/PY	3.864	0.12940	−4.605	2.745	0.710
CP_S–A_/PY	3.862	0.12946	−4.608	2.749	0.712

The value of the dipole moment of complexes in the solution phase shows that the dipole moment of the complexes has increased in going from the gas phase to the solution phase. Therefore, the dipole moment of the complexes increases after dissolution. Examination of the results in Table 3 show that the values of *E*_g_ and *η* for CP_S–A_/PY and CP_S–G_/PY complexes is less those for than CP_S–S_/PY complex; in contrast, the values of *S*, *ω*, and Δ*N*_max_ for CP_S–A_/PY and CP_S–G_/PY complexes is more than those for CP_S–S_/PY complex. These results are consistent with the gas phase results. Comparison of the solution-phase data shows that in the CP_S–S_/PY complex, the energy gap, chemical hardness, chemical potential, electrophilicity index, and Δ*N*_max_ decreased, while softness increased compared to the gas phase. The opposite trend is observed for the CP_S–A_/PY and CP_S–G_/PY complexes.

### NBO, AIM, ELF, and RDG analysis

In this work, the electron density transfers are investigated using NBO analysis. The results of the NBO analysis at the M06-2X/6-31G(d,p) level of theory are reported in Table 1. Non-covalent interactions between species lead to changes in the sum of their atomic charges. After complexation, the sum of the atomic charges of CPs and drug atoms changes, indicating that charge transfer (CT) occurs between the PY and CP. The charge transfer can be defined as the sum of atomic charges on a drug. The results show that, in all complexes, the charge transfers occur from the CP to PY. The amount of charge transfer in CP_X–Y_/PY complexes is more than that of CP_X–X_/PY complexes. This result is consistent with the higher interaction energy and higher affinity of CP_X–Y_/PY complexes with the drug than CP_X–X_/BNNT complexes. Investigations show that an alternating sequence of amino acids increases the charge transfer between the drug and the CP. In the CP_X–Y_/PY complexes, strong interactions between the drug and the cyclic peptide enhance the charge transfer from the cyclic peptide to the drug. In each class of CP_X–Y_/PY structures, a quantitative correlation is observed between the interaction energy and the charge transfer rate, such that structures with higher interaction energy show higher charge transfer rates. This trend indicates that increasing the interaction strength enhances electron redistribution, which is expected to improve the stability of the drug–carrier complex. Small values of charge transfer for non-covalent interactions indicate that no covalent bond is formed and the reversible nature of the carrier–drug interaction is maintained. Also, the data trend shows that a small increase in charge transfer is positively correlated with the enhancement of the interaction energy (Δ*E*_int_).

**Fig. 7:**
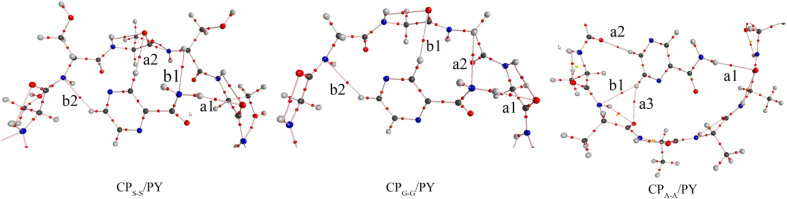
Molecular graphs for CP_X–X_/PY complexes at the M06-2X/6-31G(d,p) level of theory.

The quantum theory of atoms in molecules (QTAIM) was used to determine the nature of interactions. In this study, to determine the nature of the interactions between cyclic peptides and the PY drug, AIM analysis was performed at the M06-2X/6-31G(d,p) level of theory. The molecular graphs indicating the bond critical points (BCPs) and bond paths for CP_X–X_/PY complexes are shown in Fig. 7. In all complexes, the molecular graphs represent additional critical points in the intermolecular regions.

The values of electron density, *ρ*(*r*), the electron density Laplacian, ∇2*ρ*(*r*), and total electronic energy density, *H*(*r*), at O⋯H and N⋯H bond critical points (BCPs) of complexes are reported in Table 4.

**Table 4:** Calculated BCP data (au) for CP_X–X_/PY and CP_X–Y_/PY complexes at the M06-2X/6-31G(d,p) level of theory

Bond	*ρ*(*r*)	∇^2^*ρ*(*r*)	*H*(*r*)	*ρ*(*r*)	∇^2^*ρ*(*r*)	*H*(*r*)	*ρ*(*r*)	∇^2^*ρ*(*r*)	*H*(*r*)
	**CP**_**A**_ **_–_**_**A**_ **/PY**			**CP**_**G**_ **_–_**_**G**_ **/PY**			**CP**_**S**_ **_–_**_**S**_ **/PY**		
(O⋯H)_a1_	0.0105	0.0336	0.0077	0.0192	0.0583	0.0157	0.0194	0.0594	0.0160
(O⋯H)_a2_	0.0096	0.0304	0.0065	0.0100	0.0327	0.0068	0.0102	0.0331	0.0070
(O⋯H)_a3_	0.0058	0.0204	0.0034	—	—	—	—	—	—
(N⋯H)_b1_	0.0081	0.0248	0.0048	0.0063	0.0197	0.0035	0.0065	0.0199	0.0036
(N⋯H)_b2_	—	—	—	0.0081	0.0256	0.0049	0.0077	0.0248	0.0046

	**CP**_**A–G**_ **/PY**			**CP**_**G–A**_ **/PY**			**CP**_**S–G**_ **/PY**		
(O⋯H)_a1_	0.0106	−0.0085	0.0078	0.0195	0.0599	0.0161	0.0197	0.0599	0.0162
(O⋯H)_a2_	0.0094	−0.0074	0.0063	0.0099	0.0325	0.0067	0.0104	0.0339	0.0072
(O⋯H)_a3_	0.0059	−0.0052	0.0035	—	—	—	—	—	—
(N⋯H)_b1_	0.0082	−0.0063	0.0049	0.0061	0.0189	0.0033	0.0069	0.0213	0.0038
(N⋯H)_b2_	—	—	—	0.0079	0.0251	0.0048	0.0079	0.0254	0.0047

	**CP**_**A–S**_ **/PY**			**CP**_**G–S**_ **/PY**			**CP**_**S–A**_ **/PY**		
(O⋯H)_a1_	0.011	0.03516	0.00833	0.0195	0.0588	0.0159	0.0196	0.0588	0.0159
(O⋯H)_a2_	0.009	0.02963	0.00622	0.0104	0.0336	0.0072	0.0101	0.033	0.0069
(O⋯H)_a3_	0.005	0.0194	0.00316	—	—	—	—	—	—
(N⋯H)_b1_	0.008	0.0244	0.00482	0.0067	0.0205	0.0037	0.0061	0.0189	0.0033
(N⋯H)_b2_	—	—	—	0.0079	0.0253	0.0047	0.008	0.0255	0.0048

The results show that the electron densities at O⋯H interactions are greater than those of at N⋯H ones that are in agreement with the smaller O⋯H distance in comparison with the N⋯H ones. Thus, it is predicted that in all complexes, O⋯H interactions are stronger than N⋯H ones. Comparison of *ρ*(*r*) values shows that the *ρ*(*r*) value corresponding to the shortest interaction (O⋯H)_a_ in CP_X–Y_/PY complexes is higher than its value in CP_X–X_/PY complexes. This result is consistent with the shorter distance of this interaction in CP_X–Y_/PY complexes compared to CP_X–X_/PY complexes. The values of ∇^2^*ρ*(*r*) and *H*(*r*) at hydrogen bond critical points in all the complexes are positive. Therefore, values of ∇^2^*ρ*(*r*) and *H*(*r*) indicate that all H-bonds have electrostatic nature.

**Fig. 8:**
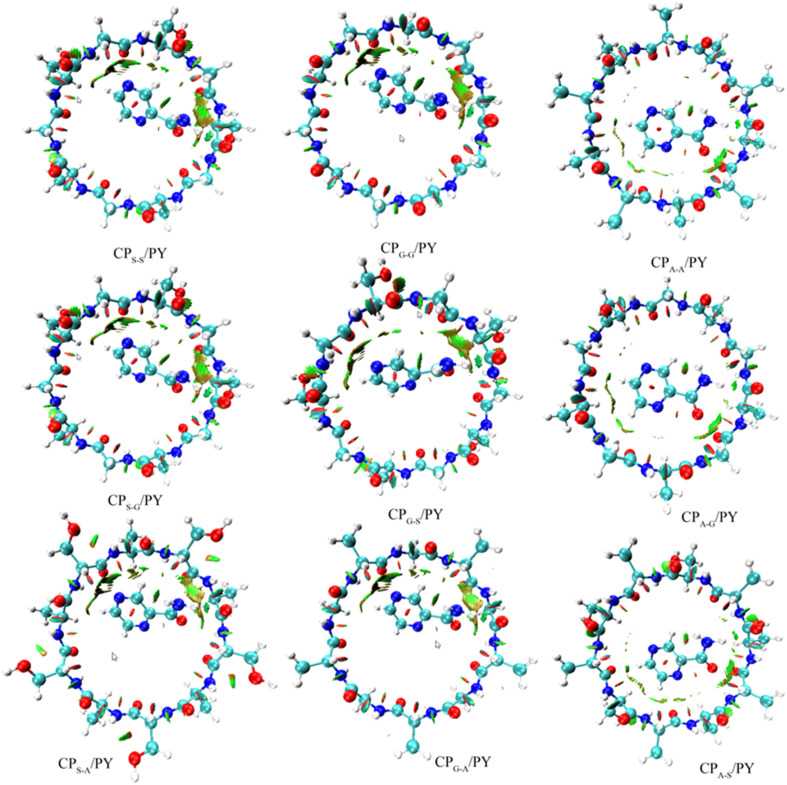
The RDG map for the optimized complexes.

The RGD analysis is used to investigate the strength of interactions in intermolecular regions. The reduced density gradient (RDG) is a scalar field of the electron density (*ρ*) that can be defined as:8
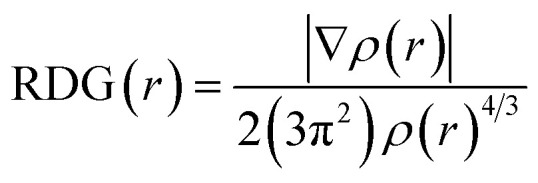
where *ρ*(*r*) and ∇*ρ*(*r*) are the electron density and its first derivative, respectively.^59^ In these diagrams, the attractive, van der Waals, and repulsive interactions are marked with blue, green, and red colors, respectively. The RDG plots for the optimized complexes are displayed in Fig. 8.

**Fig. 9:**
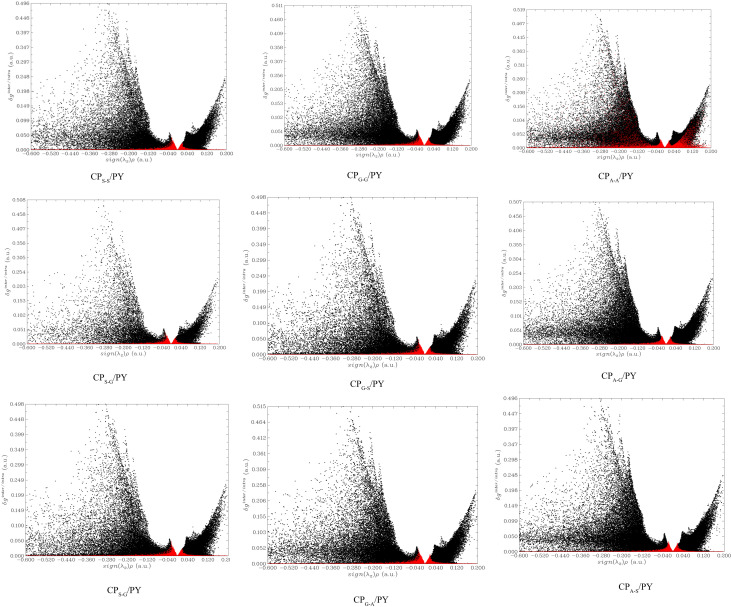
Independent Gradient Model (IGM) of PY on CP_X–X_/PY and CP_X–Y_/PY complexes.

In RDG plots, the areas between the drug and the cyclic peptides are mainly marked with green and brown color isosurfaces, which show that the interactions are of the non-covalent and vdW type. van der Waals interactions refer to weak non-covalent interactions, including hydrogen bonding, with electrostatic dominance. These non-covalent interactions are confirmed by the independent gradient model (IGM) and *δ*g descriptive function. The IGM analysis allows the separation of the *δ*g as *δ*g^inter^ and *δ*g^intra^, which solely reflect the contribution to *δ*g due to inter-fragment (non-covalent) and intra-fragment (covalent) interactions, respectively. According to the IGM-*δ*g scattered map of different complexes, inter-fragment or intra-fragment interactions are shown in Fig. 9. The red and black scattered points correspond to *δ*g^inter^ and *δ*g^intra^ fragment interactions, respectively. In the region where sign(λ2)*ρ* is about −0.04, it can be seen that the *δ*g^inter^ has a remarkable peak (with a height of nearly 0.05), which implies the presence of hydrogen bonds.

**Fig. 10:**
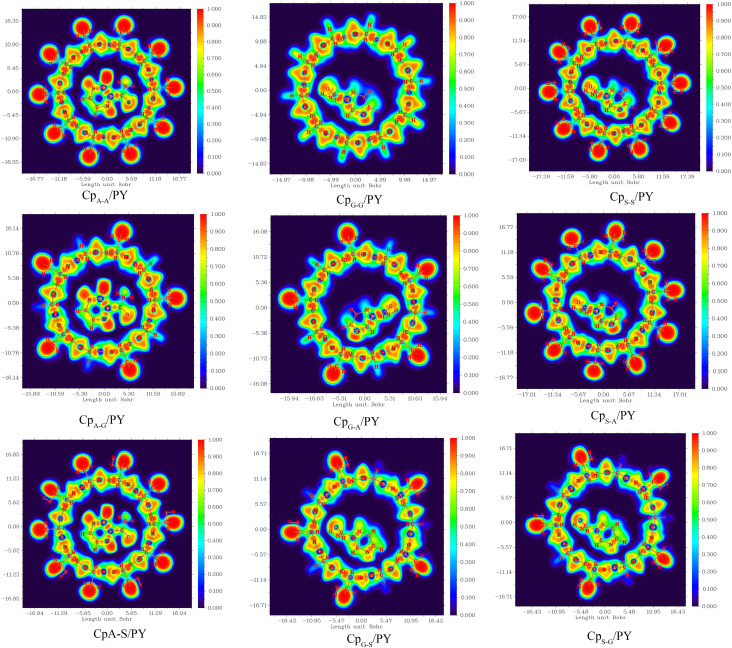
ELF maps of the interaction of the PY drug with cyclic peptides.

Subsequently, ELF (electron localization function) maps for all complexes were plotted using Multiwfn and are shown in Fig. 10.

This map represents the homogeneous Jellium-like electron gas model; the values are normalized between 0.0 and 1.0. The red regions indicate extreme localization (value 1.0). The green regions indicate a free electron gas behavior (value 0.5), and the blue regions indicate non-localization (value 0.0). The presence of these colored regions between the drug and the cyclic peptide indicates electron localization between the drug and the cyclic peptide. Fig. 10 shows relatively strong electron localization between the NH_2_CO group of the drug and the cyclic peptide ring (yellow color). This indicates that the interactions are stronger in this region.

The results of this study provide an electronic and structural basis for understanding the effect of amino acid sequences in cyclic peptides on the interaction with pyrazinamide. Therefore, this is the first step towards the rational design of drug carriers. In the next step, other properties of the structures, including thermal stability, toxicity, drug release, membrane permeability, and bioavailability, should be evaluated in *in vivo* studies.

## Conclusion

This research focuses on investigating the interaction of the anti-tuberculosis drug PY with the cyclic decapeptides of glycine (G), alanine (A), and serine (S) and their binary alternating sequences at the M06-2X/6-31G(d,p) level of theory in the gas and solution phases. So we are dealing with two types of complexes CP_X–X_/PY(CP_S–S_/PY, CP_G–G_/PY, and CP_A–A_/PY) and CP_X–Y_/PY(CP_S–A_/PY, CP_S–G_/PY, CP_G–S_/PY, CP_G–A_/PY, CP_A–G_/PY, and CP_A–S_/PY). In the obtained complexes, two types of hydrogen bonds, O⋯H and N⋯H, were observed. It seems that hydrogen bonds formed between the drug and cyclic peptides play an important role in the stability of the complexes. Comparison of the two types of hydrogen bonds in the complexes shows that the O⋯H hydrogen bonds are shorter than the N⋯H bonds. On the other hand, the O⋯H interactions observed in CP_X–Y_/PY complexes are shorter than those in CP_X–X_/PY complexes. Hence, in CP_X–Y_/PY complexes, the absolute value of *E*_HB_(|*E*_HB_|) related to an O⋯H hydrogen bond is greater than that of CP_X–X_/PY complexes. Examination of the energy results shows that the affinity of CP_X–Y_/PY complexes for drug interaction is higher than that of CP_X–X_/PY complexes. Thus, it is predicted the interactions in CP_X–Y_/PY complexes are stronger than in CP_X–X_/PY complexes. The hydrogen interactions in the complexes are of the van der Waals type. Therefore, the PY drug can physically interact with the cyclic peptides. The value of the dipole moment for CP_X–Y_/PY complexes is more than that for CP_X–X_/PY complexes, indicating that the polarity of CP_X–Y_/PY complexes is more than that of CP_X–X_/PY complexes. The energy gap values for the CP_A–A_/PY and CP_S–S_/PY complexes are more than those for the CP_X–Y_/PY complexes. Therefore, the affinity of CP_A–A_/PY and CP_S–S_/PY complexes to the PY drug is less than that of their corresponding CP_X–Y_/PY complexes. This result is also observed for the most stable complex in the solution phase. The dipole moment value of the complexes in the solution phase has increased compared to that in the gas phase. In all complexes, the charge transfer takes place from the cyclic peptide to the drug. The amount of charge transfer in CP_X–Y_/PY complexes is more than that in CP_X–X_/PY. Structures with higher interaction energy show higher charge transfer rates. Based on the AIM analysis, the electron density *ρ*(*r*) at O⋯H BCPs for CP_X–Y_/PY complexes is more than that of the CP_X–X_/PY complexes, which corresponds to the higher interaction energy in these structures. This result is in agreement with the shorter O⋯H hydrogen bond distances in the CP_X–Y_/PY complexes compared to CP_X–X_/PY ones. The nature of the O⋯H and N⋯H interactions in all complexes is electrostatic. Finally, the alternating sequence of amino acids in cyclic peptides increases the stability of the structures and improves their properties. Hence, the affinity of CP_X–Y_/PY complexes for interaction with PA is stronger than that of CP_X–X_/PY complexes.

## Conflicts of interest

All authors declare that they have no conflicts of interest.

## Data Availability

The datasets generated during the current study are available upon request from the corresponding author.
